# Mechanistic models position ceritinib as a nuclear integrity disrupting therapy in pediatric liver tumors

**DOI:** 10.1186/s13046-025-03535-z

**Published:** 2025-09-18

**Authors:** Salih Demir, Thomas Kessler, Alina Hotes, Beate Häberle, Eiso Hiyama, Tomoro Hishiki, Emilie Indersie, Sophie Branchereau, Christian Vokuhl, Mathurin Dorel, Hans Lehrach, Bodo Lange, Stefano Cairo, Roland Kappler

**Affiliations:** 1 Department of Pediatric Surgery, Dr. Von Hauner Children’s Hospital, LMU University Hospital, LMU Munich, Lindwurmstr. 2a, Munich, 80337 Germany; 2 Alacris Theranostics, Berlin, Germany; 3 Natural Science Center for Basic Research and Development, Hiroshima University, Hiroshima, Japan; 4 Department of Pediatric Surgery, Chiba University Graduate School of Medicine, Chiba, Japan; 5 XenTech, Evry, France; 6 Bicêtre Hospital, AP-HP Paris Saclay University, Paris, France; 7 Institute of Pathology, University Hospital Bonn, Bonn, Germany; 8 Max-Planck-Institute for Molecular Genetics, Berlin, Germany; 9 Champions Oncology, Inc., Rockville, MD USA

**Keywords:** Mechanistic models, Pediatric liver cancer, Therapeutic strategy, Ceritinib, Nuclear pore complex

## Abstract

**Background:**

Pediatric liver tumors with high-risk features pose therapeutic challenges, necessitating the development of more targeted and effective treatment strategies. Computational modeling of virtual patients and in silico drug response simulations, based on properly trained mechanistic models, is a powerful strategy to predict new treatment options. We aimed to leverage patient-specific mechanistic cell models to identify therapeutic alternatives for pediatric patients with high-risk liver tumors.

**Methods:**

We generated digital twins of high-risk pediatric liver tumor patients by integrating clinical, genetic, and transcriptomic data and performed computational drug response simulations using mechanistic models. We validated the therapeutic potential of ceritinib in patient-derived xenograft models both in vitro and in vivo and used fluorescence microscopy-based imaging for functional analyses.

**Results:**

Mechanistic models trained with digital twins of high-risk pediatric liver tumor patients identified ceritinib as the most effective treatment option through iterated in silico drug response simulations. Validation on a comprehensive drug-testing platform demonstrated that ceritinib, unlike other ALK receptor tyrosine kinase inhibitors with lower prediction scores, inhibited tumor growth by targeting non-canonical kinases. Mechanistically, ceritinib suppressed expression of nucleoporins, essential components of the nuclear pore complex overexpressed in pediatric liver tumors, consequently leading to the disruption of nuclear membrane integrity, perinuclear accumulation of mitochondria, production of reactive oxygen species, and induction of apoptosis. In patient-derived xenograft mouse models, ceritinib reduced tumor burden and extended survival by promoting cell death.

**Conclusion:**

This study demonstrates the successful application of mechanistic models on virtual patients to position ceritinib as a promising therapeutic agent for high-risk pediatric liver tumors, highlighting its impact on key kinases implicated in tumor aggressiveness and its ability to compromise nuclear integrity.

**Supplementary Information:**

The online version contains supplementary material available at 10.1186/s13046-025-03535-z.

## Background

Hepatoblastoma (HB) is the most common primary hepatic malignancy in children, typically presenting before the age of five years [1]. Over the past four decades, the survival rate for HB patients has significantly improved, driven by progress in surgical techniques, the introduction of orthotropic liver transplantation, and the refinement of chemotherapy regimens [2]. Recent studies report an overall survival rate of more than 80% for standard-risk patients when treated with a combination of neoadjuvant cisplatin and surgery [3]. However, a remaining group of patients demonstrates poor response to the intensified high-risk protocol, which includes alternating cycles of cisplatin and doxorubicin, and is associated with poor clinical outcome [4]. Known high-risk features include metastatic disease, vascular invasion, extrahepatic tumor spread, large pre-treatment extent of tumor (PRETEXT) 4 tumors, and age 5 years and older [5]. Additionally, hepatocellular carcinoma (HCC) and HB tumors with HCC-like features (TLCT), which usually occur in older children and adolescents, have been associated with a similar poor prognosis [6–8]. Current protocols for high-risk patients use aggressive chemotherapy regimens that lead to severe long-term side effects in pediatric patients [9]. Given these challenges, there remains an urgent need to explore novel therapeutic strategies and to identify actionable molecular targets for the treatment of pediatric liver tumors with high-risk features.

Most cancer treatment strategies continue to rely on empirical methods to determine effective therapies for the general population. However, these approaches often fail to account for individualities and necessities of patients, leading to limited success rates [10]. Adopting personalized therapies that address intertumoral heterogeneity in treatment response could enhance patient outcomes in managing the disease. Implementing advanced computational tools, such as in silico prediction systems, has demonstrated feasibility in constructing individualized patient models. These models, based on an elaborated understanding of relevant biological pathways, are highly effective in proposing new agents for targeted therapy [11]. Mechanistic models like ModCell™ leverage the “digital twin” concept, creating a virtual twin of each tumor using individual omics data to optimize treatment strategies by predicting drug responses [11–13]. Additionally, ModCell™ can facilitate drug repositioning by virtually simulating drug exposure and generating quantitative prediction scores to find the most suitable therapeutic agents, thus accelerating the determination of drug response without compromising ongoing treatments [11–13]. Based on transcriptomic sequencing data, ModCell™ has been shown to accurately predict the effect of drugs and their combinations in silico [14].

In this study, we employed clinical, genetic, and transcriptomic data from pediatric liver tumors with clinically challenging features to identify promising drugs using the ModCell™ computational prediction tool. We validated effective inhibition of HB tumor cells by ceritinib on a comprehensive drug-testing platform and in the in vivo setting. We could trace the primary response to ceritinib to the disruption of nuclear membrane integrity through the downregulation of nucleoporins, leading to apoptosis in high-risk HB.

## Methods

### Patient cohort

A total of 13 pediatric liver tumor samples were collected from patients undergoing surgical resection in our department, with corresponding normal liver tissue available from 8 of these patients. Written informed consent was obtained from all participants, and the study protocol was approved by the Ethics Committee of the Ludwig-Maximilians-University, Munich. A summary of patient characteristics is provided in Supp. Table 1.

Molecular baseline analysis was conducted on DNA and RNA extracted from snap-frozen tumor tissue as previously described [6]. Specifically, DNA was analyzed for mutations in *CTNNB1*, *NFE2L2*, and the *TERT* promoter by Sanger sequencing and the 16-gene signature was determined by quantitative PCR and BRB ArrayTools [6].

Expression analysis of ceritinib-targeted candidate genes in hepatoblastoma patients was accomplished using the GSE131329 dataset of the Japanese Pediatric Liver Tumor trial JPLT-2 [15] publicly accessible from the R2 genomics analysis and visualization platform (http://r2.amc.nl).

### RNA sequencing

Total RNA was extracted from fresh-frozen tumor and adjacent normal liver tissues or patient-derived xenograft (PDX) cell lines as described previously [16]. RNA libraries were prepared from 1 µg of total RNA using the TruSeq stranded mRNA kit (Illumina, San Diego, CA, USA) and sequenced on a HiSeq2500 system (Illumina) as 100 bp paired-end runs generating ~ 50 million mapped reads per sample. Alignment against the human genome assembly hg19 (GRCh37) and UCSC gene annotation as well as quantification of reads mapping to annotated genes were carried out using STAR aligner v 2.4.2a and HTseq-count v0.6.0, respectively. Read counts from RNA-sequencing data were normalized and analyzed for differential gene expression between patient-vs-normal or treated-vs-untreated cells using the Bioconductor package DESeq2 [17].

Genes that were downregulated upon ceritinib exposure by less than twofold with a *p*-value < 0.05 were analyzed using the ShinyGO v.0.80 (http://bioinformatics.sdstate.edu/go/) database to identify altered gene ontology (GO) terms and enriched KEGG pathways. Moreover, a pre-ranked gene expression list according to the fold-increase was analyzed by applying KEGG-Medicus gene set of the human molecular signature database (MSigDB v2023.2. Hs) via gene set enrichment analysis tool (GSEA) (https://www.gsea-msidb.org). Enriched pathways in tumor patients in comparison to the normal liver were determined by employing Hallmark gene set of MSigDB.

### Computational drug prediction and in silico testing by ModCell™

ModCell™ is based on an ordinary differential equations (ODEs) systems biology platform model (an sbml version can be downloaded from: [12]) for the simulation, prediction and drug screening of complex molecular systems covering 47 signaling pathways, 720 genes and > 6,000 biochemical species interconnected by > 9,000 ODE reactions [11–13]. We individualized the ModCell™ ODE model for each pediatric liver tumor patient of our cohort based on expression data of tumors and corresponding normal tissue by initiating a distinct synthesis parameter for each gene-representative in the model. We then simulated the ODE until a steady-state was reached for all species contained in the model, either with or without the concurrent simulation of molecularly targeted drugs. The action of molecularly targeted drugs was reflected through the kD-parameter associated with the specific drug-target interaction equation, as obtained from a comprehensive review of relevant literature (unpublished data). For each patient we reiterated these steady-state simulations 1,000 times with different sets of parameters as previously described for the Monte Carlo approach [12, 18]. Then, the relative abundance of readout species was calculated for the median of Monte Carlo runs at baseline and for different concentrations of model drugs, so that a differential activity of readout species could be calculated for each in-silico drug perturbation across simulated steady states. Based on our transcriptomic data, we selected the model species “nuclear MYC:MAX complex” and “active Caspase-3” as suitable readouts for positive and negative phenotypic effects of cell viability in tumor simulations, respectively. For a given patient and perturbation, the sum of the simulated concentration of these species was calculated and compared to the concentration in a control simulation. From our genetic data, we selected “CTNNB1” as the most common and significant driver for model activation. With these settings, an in silico drug response screen was performed, modeling 20 molecularly targeted drugs in a concentration range of 1e-4 to 1e4 nM. The ModCell™ data analysis and visualization pipeline were used for result interpretation. Predicted maximal tumor growth inhibition (Emax) was calculated for individual drugs, with an Emax cutoff of 50% indicating a potentially effective drug in a patient. For these drugs, the concentration at the half maximum inhibition value (EC50) was calculated based on individual drug-response curves across the simulated concentration range as presented. In order to calculate drug response levels, we used the normalized growth rate inhibition (GR) approach [19], which enables computing drug sensitivity without the issue of unequal division rates that can confound the results and make it difficult to compare growth rates between different cell lines. The normalized GR approach yields per-division metrics for drug potency (GR50) and efficacy (GRmax) that are analogous to the more familiar IC50 and Emax values.

### Drug testing platform

The in vitro drug testing platform [20] comprised of three HB cell lines (HuH6, HepG2, HepT1) and two HCC cell lines (Hep3B, HuH7), along with seven HB PDX tumor cell lines (PDX214, PDX243, PDX282, PDX295, PDX303, PDX344, PDX346). Additionally, primary adult (HDFa) and neonatal (HDFn) human dermal fibroblasts, epidermal keratinocytes, human bronchial epithelial cells (BEAS-2B) as well as embryonal kidney epithelial cells, served as non-cancerous healthy controls. Detailed information on cell lines is listed in Supp. Table 2.

Models in the platform were seeded as 5 × 10^4^ cells in 96-well plates, as two replicates, and then exposed to 10 increasing concentrations of the compound-of-interest, ranging from 5 nM to 100 µM in 1:3 constant dilution ratios, for 48 h. MTT (3-(4, 5-dimethylthiazol-2-yl)−2, 5-diphenyltetrazolium bromide) (Carl Roth, Karlsruhe, Germany) viability assay were carried out as recommended by the supplier. Nonlinear regression curves of drug response versus the inhibitors were plotted and the sensitivities were determined by calculating half-maximal inhibitory concentrations (IC50) and area under the curve (AUC) values, using GraphPad Prism 8 (GraphPad Software, San Diego, CA). Z-scores were calculated with the formula: [(Individual AUC value)-(mean AUC)]/(standard deviation). Therapeutic window scores (TWS) were calculated by using the formula: (mean AUC of healthy controls)/(mean AUC of tumor models). Detailed information on compounds used is listed in Supp. Table 3.

### Western blot analysis

Whole cell lysates were isolated and prepared as previously described [20]. For phospho-proteins, cell lysis buffer was supplemented with 1 × Halt™ Phosphatase Inhibitor Cocktail (Thermo Fisher, Waltham, MA). Proteins (25 µg) were denatured at 70 °C for 10 min, separated on a 4–20% gradient gel (Thermo Fisher) and transferred to nitrocellulose membranes with 0.2 µm pore size (BioRad, Hercules, CA). The membranes were blocked in 5% milk powder (Carl Roth) diluted in Tris buffer saline with 0.1% Tween 20 (Carl Roth), and probed with corresponding 1:1,000 primary antibody dilutions overnight at 4 °C. Following 1 h incubation with the respective secondary antibodies, the proteins were detected using enhanced chemiluminescence detection reagent (Amersham Biosciences, Amersham, UK) and imaged with the ChemiDoc XRS + system (Bio-Rad). Detailed information on antibodies is provided in Supp. Table 4.

### Detection of long- and short-term and three-dimensional growth

Short-term growth, demonstrated by cell proliferation, was assessed using the Click-iT EdU cell proliferation kit (Thermo Fisher) following the manufacturer's instructions. Briefly, 2 × 10^5^ cells were labeled with 100 µM ethynyl deoxyuridine (EdU) and then treated with either 250 nM ceritinib or DMSO for 24 h. The proliferation was calculated by the relation of Alexa Fluor 555 azide stained-EdU-positive nuclei to Hoechst 33342 (Thermo Fisher)-counterstained nuclei. Microscopy images were captured using the EVOS M7000 imaging system (Thermo Fisher).

Long-term growth of the tumor cells was measured using a colony formation assay, where 2 × 10^3^ cells per well were seeded in 6-well plates and exposed to either DMSO or 250 nM ceritinib for 10 days. After colony formation, cells were stained with 0.5% crystal violet (Sigma-Aldrich, St. Louis, MO) in 20% methanol for 2 h. Images of the colonies were captured using the GelJet Imager (INTAS, Göttingen, Germany) and the EVOS M7000 (Thermo Fisher) for overview and detailed examination, respectively.

For three-dimensional growth, 1 × 10^3^ cells in 100 µl/well were seeded into ultra-low attachment round-bottom 96-well plates (Corning, Corning, NY) and incubated for 5 days to allow spheroid formation. Once spheroids were established, they were treated with either 250 nM ceritinib or DMSO, and images were captured on days 2 and 6. All microscopy images were taken using the EVOS M7000 (Thermo Fisher).

### Wound healing assay

Cell migration was evaluated by wound healing assay. Cells were seeded in a 6-well plate at a density of 1 × 10^6^ cells per well and allowed to grow to 90% confluence. A scratch was made in the center of each well using a 1-mL pipette tip, ensuring the tip was perpendicular to the well bottom to create a consistent gap. The wells were then washed with fresh medium to remove any detached cells. The cells were treated with either DMSO or 250 nM ceritinib in 2 mL of fresh medium for 2 days and microscopy images taken every 24 h using EVOS M7000 (Thermo Fisher). The rate of wound closure was calculated using the formula: [(initial wound gap)-(wound gap on day 1) × 100/(initial wound gap)].

### Detection of nuclear integrity irregularities

Integrity of nuclear membrane was detected by Vybrant Dil live staining according to the manufacturer’s instructions (Thermo Fisher). Briefly, 1 × 10^6^ cells were seeded after being resuspended in 1 ml serum-free medium and incubated with 20 µl of dye solution in the dark for 30 min. 2 h post-seeding, Vybrant Dil-labelled cells were exposed to DMSO, 250 nm ceritinib, 1 µM rapamycin, 1 µM trametinib, 1 µM capivasertib, or 1 µM defactinib for 24 h. Detailed information on compounds used is listed in Supp. Table 3.

Nuclear circularity and nuclei area were detected by Hoechst 33342 staining of DMSO or 250 nM ceritinib treated cells after 24 h. Circularity index (CI) was calculated by [4π x (area)]/(perimeter)^2^ formula. All microscopy images were captured by EVOS M7000 (Thermo Fisher).

### Detection of reactive oxygen species (ROS)

Reactive oxygen species detection assay kit (Abcam, Cambridge, UK) was used for detecting cellular ROS levels following the manufacturer`s instructions. 5 × 10^5^ cells were treated with DMSO or 250 nM ceritinib for 24 h, then stained with permanent 2´−7´-dichlorofluorescin diacetate (DCFDA) dye for 40 min at 37 °C. 30 µM radical initiator tert-Butyl hydroperoxide (TBHP) was used as a positive control. ROS levels were determined by both microscopy with EVOS M7000 (Thermo Fisher) and flow cytometry with LSRFortessa™ cell analyzer (Becton Dickinson Biosciences, Franklin Lakes, NJ, USA). Mean fluorescence intensity values (MFI) of DCFDA staining were calculated by FlowJo™ v10.10 (BD biosciences, Franklin Lakes, NJ, USA).

### Apoptosis assay

Apoptosis was detected by CellEvent™ Caspase-3/7 Green Detection Reagent (Thermo Fisher) according to the manufacturer’s instructions, by exposing 2 × 10^5^ cells to 250 nM ceritinib or DMSO for 24 h and detecting cells that were positive for active substrates of caspase 3 and 7 using the EVOS M7000 imaging system (Thermo Fisher).

### Mitochondria tracking

Subcellular localization of mitochondria was detected by MitoTracker Green FM dye according to manufacturer`s instructions (Thermo Fisher). In short, 2 × 10^5^ cells were expose to 250 nM ceritinib or DMSO for 24 h, then labelled with 200 nM/ml MitoTracker Green for 30 min at 37 °C. Subcellular images of mitochondria were captured by EVOS M7000 imaging system (Thermo Fisher).

### Combination assay

The synergistic potential of two-drug combinations was investigated by measuring tumor cell viability by MTT assay after exposure to pairwise combinations of cisplatin, doxorubicin and ceritinib in a 4 × 4-isobologram format for 48 h. Synergy score and two-dimensional synergy contours were obtained via the SynergyFinder + web-tool by applying the Bliss statistical reference model [21].

### Animal experiments

The preclinical study in mice was carried out by XenTech, as previously described [22], under the license for experiments on vertebrate animals (APAFIS#29,136–2,020,121,415,204,532). In brief, cryopreserved tumor pieces of the PDX model HB-282 were subcutaneously implanted in the interscapular region of 5-week-old female nude-Foxn1nu mice (ENVIGO, Gannat, France) and once tumors grown until a volume of between 75 and 288 mm^3^, mice were treated with either vehicle or 50 mg/kg ceritinib in 10% DMSO/40% PEG300/5% Tween/45% NaCl per oral administration for 5 days on and 2 days off for 3 weeks. Tumor volume (TV) was calculated by the formula: TV (mm^3^) = [(length (mm) × width (mm)^2^)/2]. All animals were weighed at tumor measurement times and monitored every day for physical appearance, behavior, and clinical changes.

Liver tumors were histologically diagnosed on hematoxylin (Carl Roth) and eosin (Sigma-Aldrich) stained cryosections of dissected tumors by a trained pathologist. For immunohistochemistry, cryosections with 5 µm-thickness were fixed with 4% paraformaldehyde, permeabilized with 0.5% Triton-X-100 and blocked in PBS-T containing 3% BSA and 0.1% glycine. Slides were then incubated with cleaved caspase-3 and Ki67 antibodies overnight and labelled with Alexa Fluor 555 anti-rabbit IgG secondary antibody (Invitrogen) on the following day. Detailed antibody information is listed in Supp. Table 4. All images were captured with EVOS M7000 (Thermo Fisher).

### Statistical analysis

GraphPad Prism 8 (La Jolla, CA, USA) was used to perform statistical analyses. Statistical significance of binary variables was determined by a two-tailed, unpaired Student’s t test with a value of *p* < 0.05 considered significant. Values with error bars shown in figures are means ± SEM unless indicated otherwise.

## Results

### Computation of virtual patient mechanistic cell models predicts ceritinib as a candidate drug for high-risk pediatric liver tumors

**Fig. 1:**
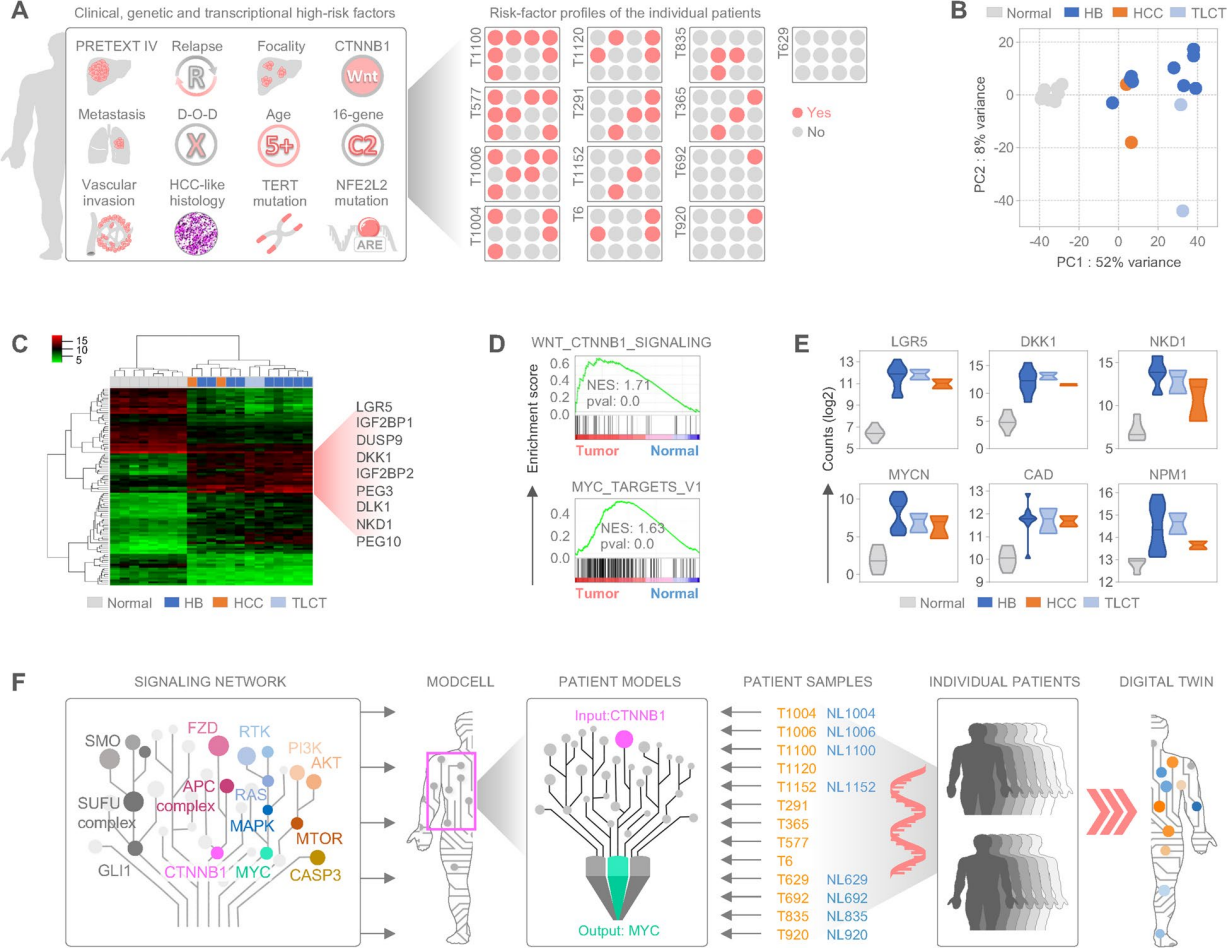
Mechanistic models using virtual twins identify ceritinib as a drug candidate for high-risk HB patients. **A** Illustrative representation of high-risk features of pediatric liver tumors (left part) and color-coded clinical and molecular characteristics of patients in the study (right part). **B** Principal component analysis displaying transcriptional proximity of samples. HB, hepatoblastoma; HCC, hepatocellular carcinoma; TLCT, transitional liver cell tumor; Normal, non-cancerous liver tissue. **C** A heatmap demonstrating the top 100 differentially expressed genes obtained from RNA sequencing of 13 liver tumor and normal liver tissue samples. Upregulated tumor-specific genes are highlighted. **D** Enrichment plots of hallmark gene sets “WNT_CTNNB1_Signaling” and “MYC_Targets_V1” calculated for 13 liver tumors versus 8 normal livers. Normalized enrichment scores (NES) and p-values (pval) are noted. **E** Violin plots displaying RNA counts of Wnt signaling genes (upper row) and MYC target genes (lower row). Middle lines demonstrate the median expression. **F** Schematic representation of the ModCell mechanistic model with implemented cellular signaling pathways, trained by CTNNB1 as input, MYC as readout, and individual patient profiles for generating digital twins

To leverage mechanistic models developed within ModCell™ [11, 13] for drug prediction in pediatric liver tumors, we assembled a cohort of 13 patients with individual high-risk disease conditions, including large PRETEXT 4 tumors, metastasis, vascular invasion, hepatocellular histology (HCC and TLCT), multifocal and recurrent disease, age over 5 years, as well as poor outcome (Fig. 1A). To identify key drivers and activated signaling pathways for the use as common activators and readouts in mechanistic models, we conducted genetic testing and RNA sequencing. We identified *CTNNB1* mutation as the most prevalent genetic alteration in 11/13 patients across all three liver tumor types (Fig. 1A). Principle component analysis (Fig. 1B) and hierarchical clustering (Fig. 1C) of expression profiles distinctly separated liver tumor samples from eight normal liver samples. Among the top 100 deregulated genes, Wnt-associated genes such as LGR5, DKK1, and NKD1, as well as the HB-related genes DUSP9, DLK1, PEG3, PEG10, IGF2BP1, and IGF2BP2 were consistently upregulated in tumor samples (Fig. 1C). Gene set enrichment analysis (GSEA) further reinforced shared enrichment of Wnt signaling genes, as well as MYC target genes (Fig. 1D). Specifically, individual gene analysis showed profound upregulation of LGR5, DKK1, and NKD1 as well as MYCN, CAD, and NPM1 across all tumors (Fig. 1E). To model molecular interactions in sufficient complexity, we used ModCell™ [11–13], a mechanistic model based on cancer-related signaling pathways such as the Hedgehog, Wnt, RAS/MAPK, and PI3K/AKT/MTOR signaling, that either enhance MYC transcriptional activity or suppress apoptosis via effector caspases (Fig. 1F). We selected “CTNNB1/Wnt activation” as a significant activator for ModCell™, and the model species “nuclear MYC:MAX complex” as a suitable readout for positive phenotypic effects on cell viability (Fig. 1F). These biological networks were then individualized based on transcriptomic data to generate digital twins of our 13 high-risk pediatric liver tumor patients.

**Fig. 2:**
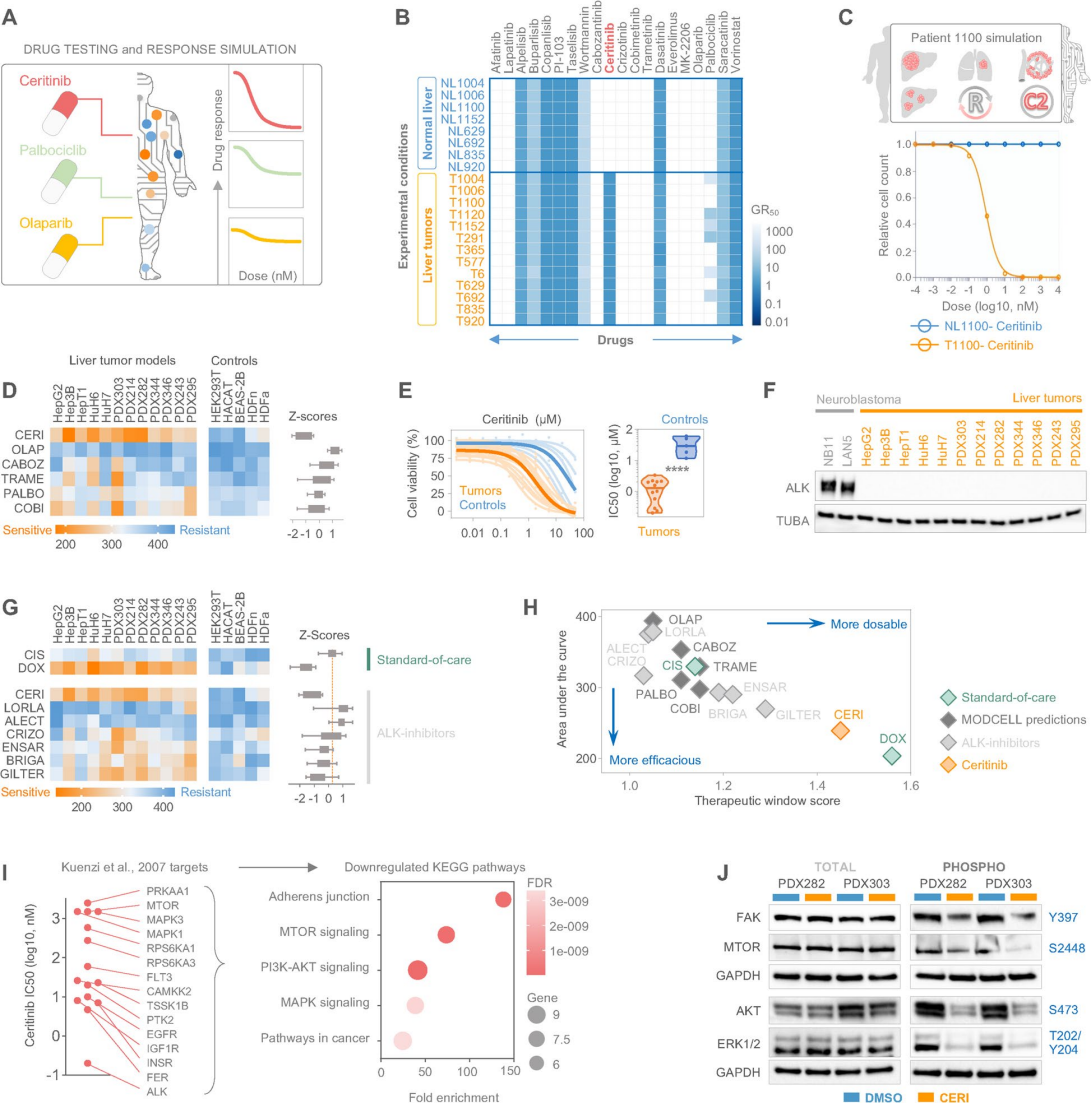
Ceritinib impedes liver tumor growth through ALK-independent kinase inhibition. **A** Prototypic in silico-drug testing and response simulation by using virtual patient cell models created via ModCell. **B** Drug response prediction heatmap using growth rate inhibition (GR50) as metric for drug sensitivity. Heatmap summarizes the approximated response towards 20 compounds for 13 liver tumor and 8 normal livers. The most discriminative drug between simulated tumor and normal liver samples, ceritinib is highlighted in red fond. **C** Simulated ceritinib drug response curves for patient 1100, either for normal control tissue (blue line) or tumor tissue (orange line). Clinical high-risk characteristics of the simulated patient are shown in the illustrative panel. **D** Heatmap of drug sensitivity values calculated from area under the curve in liver tumor cell models. CERI, ceritinib; OLAP, Olaparib; CABOZ, cabozantinib; TRAME, trametinib; PALBO, Palbociclib; COBI, cobimetinib (left). Z-scores representing the overall response range of each compound is shown in box-with-whiskers graph, in which whiskers represent 25th and 75th percentiles (right). **E** Viability curves (left) and IC50 values in violin plot (right) for liver tumor cell lines (orange) and healthy controls (blue) demonstrates sensitivity to ceritinib. Student`s t test was applied for the significance, ****p < 0.0001. **F** Western blot analysis of ALK protein in liver tumor models. Two neuroblastoma cell lines were used as ALK-expressing positive controls and alpha tubulin (TUBA) served as a loading control. **G** A heatmap showing the areas under the curve values of liver tumor cell models upon standard-of-care therapy drugs and ALK inhibitors (left). CIS, cisplatin; DOX, doxorubicin; CERI, ceritinib; LORLA, lorlatinib; ALECT, alectinib; CRIZO, crizotinib; ENSAR, ensartinib; BRIGA, brigatinib; GILTER, gilteritinib. Box-with-whiskers graph with 25th and 75th percentiles, demonstrating calculated z-scores for each compound (right). Dashed line indicates the mean z-score of cisplatin. **H** Symbol plot demonstrating all tested compounds according to their effectivity (area under the curve) and range of therapeutic window score. **I** Scatter plot showing previously published ceritinib targets [23] and their inhibition concentrations based on enzymatic assays (left). Bubble plot displaying enrichment scores of KEGG pathways upon ceritinib exposure, in which size of bubbles represent the protein number and color scheme indicates false discovery rate (FDR) (right). **J** Western blot analysis demonstrating the expression levels of total (left) and phosphorylated forms (right) of indicated kinases in two untreated (DMSO) and 250 nM ceritinib-treated (CERI) patient-derived xenograft (PDX) cell models (16 h). Blue text denotes the phosphorylation sites. Glycerinaldehyd-3-phosphat-dehydrogenase (GAPDH) served as a loading control

Next, we performed personalized drug response simulations on digital twins of individual tumor and normal liver tissues (Fig. 2A). This strategy is based on a Monte Carlo approach, in which the parameters are repeatedly sampled from specific probability distributions and used for multiple parallel simulations [18]. This produced dose–response curves for each drug across individual patient samples, covering a specific concentration range for each drug. To calculate drug response levels, we employed the normalized growth rate inhibition (GR) approach [19] and identified several drugs with strong inhibitory effects across all samples in the cohort (Fig. 2B). For ceritinib, modeling revealed a strong inhibitory effect selectively on simulated tumor samples, with minor predicted impact on normal liver tissue samples by drug simulation (Fig. 2B). A representative drug response curve from the in silico simulation for patient 1100 harboring multiple clinical high-risk criteria showed striking predicted growth inhibition to ceritinib exposure in the tumor, while sparing normal liver tissue (Fig. 2C). Notably, inclusion of mutational status, irrespective of tumor subtype and clinical parameters did not significantly alter drug predictions, thereby emphasizing ceritinib’s potential as a general therapeutic candidate for all types of pediatric liver tumors.

To validate the therapeutic efficacy of drugs predicted by ModCell™, we next determined tumor cell viability upon drug treatment using a recently developed drug testing platform comprised of five liver tumor cell lines and seven established PDX lines, most of which show Wnt activation mainly through CTNNB1 or AXIN1 mutations (Supp. Table 2), as well as five non-cancerous controls [20]. Drug sensitivities were determined by calculating the area under the curve (AUC) values from drug response curves, based on ten increasing concentrations of the selected drugs. As predicted by ModCell™, ceritinib showed a remarkable growth inhibitory effect on tumor cells, with very small AUC values, nanomolar range half-life inhibitory concentrations (IC50) and low z-scores (Fig. 2D). In contrast, ceritinib had minor inhibitory effects on the growth of non-cancerous cells, consistent with the ModCell™ prediction (Fig. 2E). Importantly, ceritinib outperformed other compounds with lower ModCell™ prediction scores, including olaparib, cabozantinib, trametinib, palbociclib and cobimetinib, confirming the drug prediction accuracy of ModCell™ (Fig. 2D). In summary, our computational approach identified ceritinib as a potential new drug for treating high-risk pediatric liver tumors.

### Ceritinib impedes multi-kinase activity in pediatric liver tumors

As ceritinib is primarily known as a selective ALK inhibitor [24], we wanted to investigate if ALK was the target in pediatric liver tumors. However, Western blot analysis revealed no detectable ALK in any of the liver tumor models (Fig. 2F), ruling out the possibility that the anti-tumor effect of ceritinib is mediated through ALK inhibition. Importantly, when we tested six other selective ALK inhibitors on our tumor model collection, we found that ceritinib was by far the most efficacious compound, comparably strong to doxorubicin and much stronger than cisplatin, the standard-of-care therapeutics for HB (Fig. 2G and H). To identify alternative molecular pathways affected by ceritinib, we examined previously published non-canonical targets of ceritinib [23]. Pathway enrichment analysis revealed that ceritinib drastically downregulated adherens junction, mechanistic target of rapamycin kinase (MTOR), PI3K-AKT, and mitogen-activated protein kinase (MAPK) signaling pathways (Fig. 2I). Moreover, Western blot analysis validated these findings, as ceritinib exposure significantly decreased the phosphorylation of key components of these pathways, namely focal adhesion kinase (FAK), MTOR, AKT, and ERK1/2 proteins. This suggests that ceritinib interferes with multiple kinases beyond its primary target in pediatric liver tumors and abrogates the respective signaling pathways by impairing their activation (Fig. 2J).

### Ceritinib downregulates nucleoporins, leading to dysregulation of nuclear pore complex

**Fig. 3:**
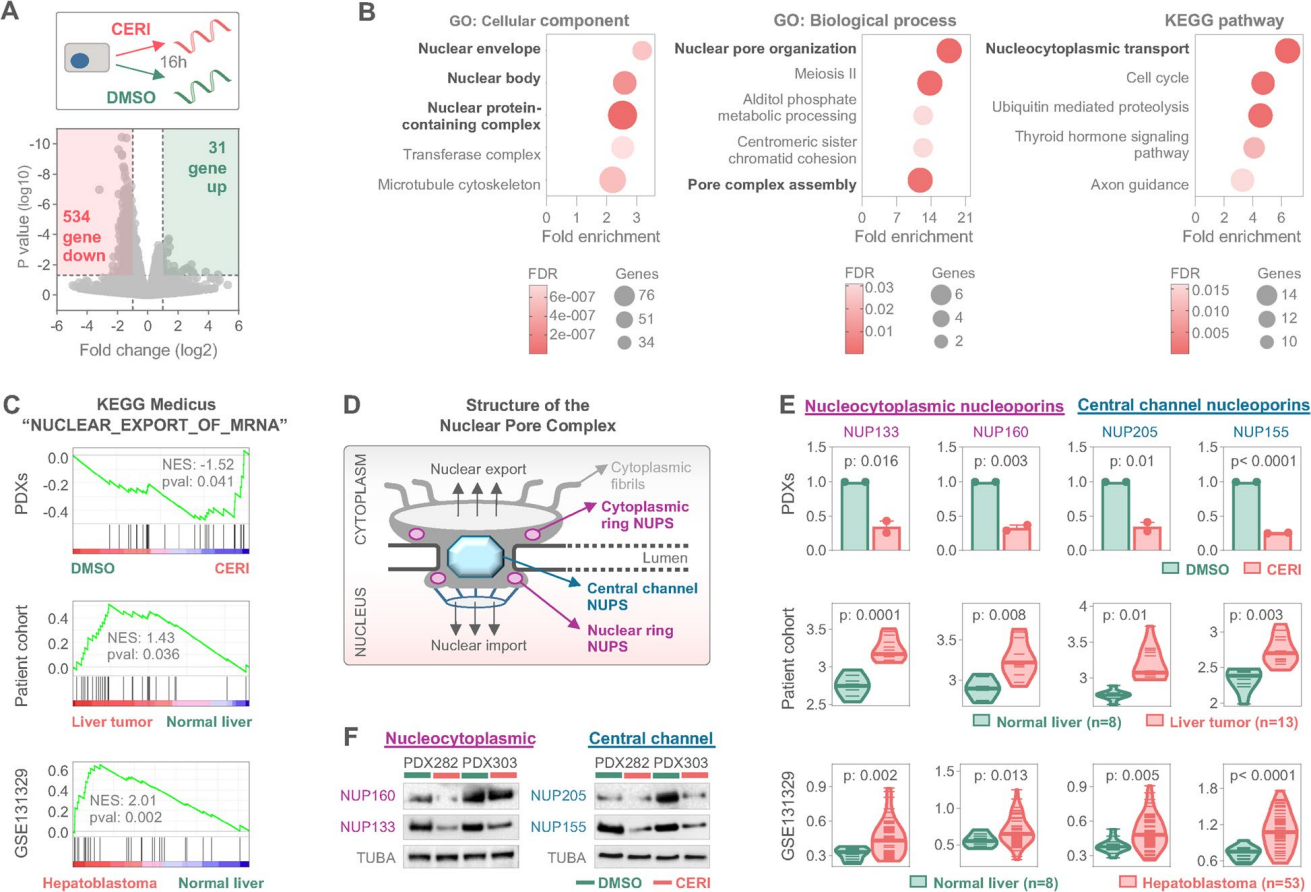
Ceritinib causes transcriptional suppression of nucleoporins. **A** RNA sequencing of two HB patient-derived xenograft (PDX) models upon DMSO or 250 nM ceritinib (CERI) exposure for 16 h. Volcano plot of differentially expressed genes demonstrating significantly up- (green) and down-regulated (red) genes. Dashed lines represent log2 fold increase and *p* < 0.05 significance thresholds. **B** Pathway enrichment analysis of down-regulated genes using gene ontology (GO) and Kyoto encyclopedia of genes and genomes (KEGG). Top scoring terms are given as separate bubble plots, with the size of the bubble representing the enriched gene number, distance from y-axis showing the fold enrichment, and color scale corresponding to false discovery rate (FDR); cellular components (CC); biological processes (BP). **C** Gene set enrichment analysis of pre-ranked RNA sequencing data based on fold increase, showing the negatively enriched KEGG-MEDICUS term “NUCLEAR_EXPORT_OF_MRNA” for two PDX models (top), 13 liver tumor patients (middle) and 53 HB patients from GSE131329 data set (bottom). **D** Illustrative structure of nuclear pore complex and localization of nucleoporins (NUPs) are demonstrated. **E** Relative RNA expression of the nucleoporins NUP133, NUP160, NUP155 and NUP205 in tumor cells treated with DMSO or 250 nM CERI (top). Comparative analysis of NUP expression in our patient cohort (*n* = 13) and normal liver (*n* = 8) on the transcript level (middle). RNA levels of nucleoporins in 53 hepatoblastoma patients and corresponding normal liver tissues (*n* = 14) from the GSE131329 dataset of the JPLT-2 trial (bottom). The student’s t test was applied to calculate significance. **F** Western blot analysis displaying the protein levels of NUP133, NUP160, NUP155 and NUP205 upon 16 h treatment with DMSO or 250 nM ceritinib. Alpha tubulin (TUBA) served as a loading control

To understand the molecular consequences of ceritinib exposure, we performed RNA sequencing on two high-risk PDX cell models (PDX282 and PDX303) treated either with DMSO or 250 nM ceritinib, corresponding to the IC50 concentration as determined in our drug testing platform (Fig. 2E). The comparison of the global RNA expression levels revealed 31 and 534 genes to be significantly (*p* < 0.05) induced and repressed > twofold by ceritinib treatment, respectively (Fig. 3A). Given the predominance of repressed genes, we explored enriched gene ontologies and pathways associated with ceritinib-induced transcriptional downregulation. Gene ontology analysis of cellular components and biological processes, along with KEGG pathway analysis, highlighted a significant enrichment in nuclear membrane and nucleocytoplasmic transport-related terms (Fig. 3B). To deduce key ceritinib-triggered transcriptomic changes, we employed the KEGG-Medicus dataset, which integrates health-associated KEGG networks, drugs, and disease databases. Gene set enrichment analysis revealed that ceritinib heavily mediated transcriptional downregulation of genes associated with “nuclear export of mRNA” (Fig. 3C, top). Interestingly, the negatively enriched gene set primarily consisted of nucleoporins (NUPs), essential structural components of the nuclear pore complex (NPC) that facilitate nucleocytoplasmic cargo export and import (Fig. 3D). Specifically, two major components of the outer ring complex, NUP133 and NUP160, which are symmetrically localized in both nuclear and cytoplasmic membranes and regulate nucleocytoplasmic transfer [25, 26], were significantly repressed upon ceritinib exposure (Fig. 3E, top-left). Moreover, two main NUPs of the central channel`s spoke ring complex, NUP205 and NUP155, which are crucial for nuclear envelope formation and mRNA translation [27], were also downregulated following ceritinib treatment (Fig. 3E, top-right). Notably, analysis of our high-risk tumor patient cohort, as well as a larger and independent cohort from the JPLT-2 trial [15] revealed a prominent enrichment and transcriptional upregulation of these four NUPs in tumor tissue as compared to normal liver tissue (Fig. 3C and E, middle and bottom). Importantly, Western blot analysis validated the downregulation of NUPs upon ceritinib exposure on the protein level (Fig. 3F). In summary, these data clearly indicate that ceritinib leads to dysregulation of important components of the nuclear pore complex, which might impair nuclear cargo trafficking by downregulating NUPs that are highly expressed in pediatric liver tumors.

### Ceritinib-induced nuclear membrane disruption triggers ROS accumulation and tumor cell death

**Fig. 4:**
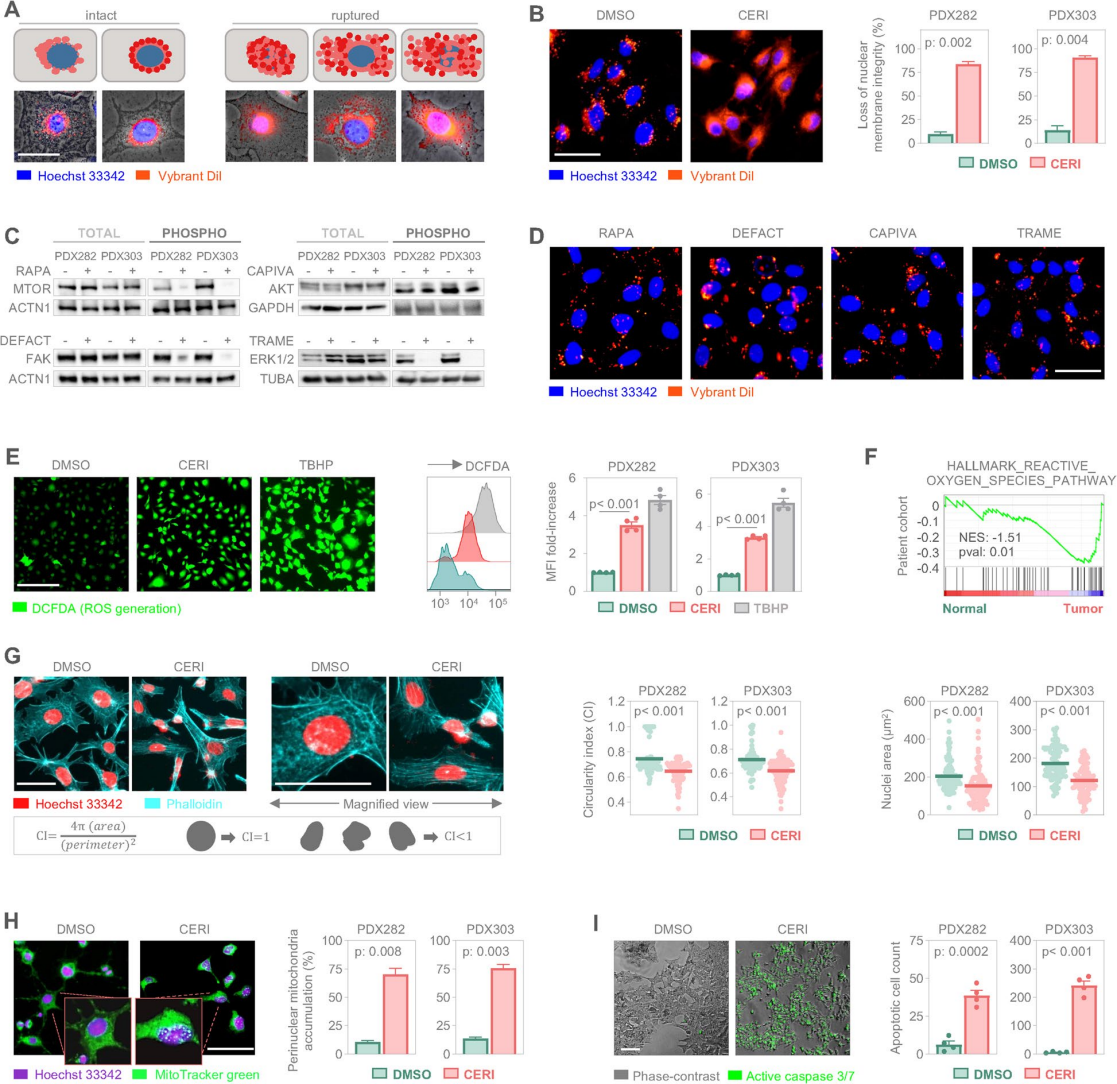
Ceritinib disrupts nuclear membrane integrity and triggers apoptosis by increasing ROS generation. **A** Illustrative (top) and microscopic (bottom) images of Vybrant Dil labeled nuclei classification based on nuclear membrane integrity. Cells with intact (left) and ruptured (right) nuclear membrane are demonstrated. Ruptured membrane leads to nuclear, cytoplasmic or nuclear and cytoplasmic leakage. **B** Representative Vybrant Dil labeled (red) tumor cells upon 24 h DMSO or 250 nM ceritinib (CERI) exposure shown in contrast to Hoechst 33424 (blue) staining (left). Bar graphs display standard error of mean (± SEM) of two independent experiments, in which at least 150 cells were counted. **C** Western blot analysis demonstrating the expression levels of total and phosphorylated forms of indicated kinases in two liver tumor models upon 16 h exposure with rapamycin (RAPA), capivasertib (CAPIVA), defactinib (DEFACT), and trametinib (TRAME). Alpha-Actinin (ACTN1), glycerinaldehyd-3-phosphat-dehydrogenase (GAPDH), and alpha tubulin (TUBA) served as loading controls. **D** Representative Vybrant Dil labeled (red) tumor cells co-labeled with Hoechst 33424 (blue) upon 24 h treatment with 1 µM of the indicated kinase inhibitors. **E** Microscopy images (left), flow cytometry histograms (middle) and mean fluorescent intensity (MFI) values of 2´−7´-dichlorofluorescin diacetate (DCFDA)-positive ROS-generating cells upon 24 h DMSO or 250 nM CERI treatment. Auto-oxidation agent tert-butylhydroperoxid (TBHP) was used as a positive control. Bar graphs represent the mean ± SEM of two independent experiments with duplicate measurements. **F** Enrichment plot of the hallmark gene set “REACTIVE_OXYGEN_SPECIES_PATHWAY” obtained by analyzing differentially expressed genes from 13 liver tumors versus 8 normal livers. **G** Representative Hoechst 33424 (red) stained nuclei (left) in contrast to phalloidin (cyan) and magnified views (right) are demonstrated. Circularity index formula and cell archetypes as silhouettes are given in the lower panel. Circularity indices (left) and nuclei areas (right) of 100 cells following 24 h exposure to DMSO or 250 nM CERI are displayed as dot plots, in which each symbol represents an individual cell and middle lines show the mean. **H** Subcellular localization of mitochondria, detected by Mitotracker green, is shown in contrast to Hoechst 33,424 stained nuclei (purple). Magnified cell views are given in the insets. Bar graphs represent mean ± SEM of two independent experiments, in which at least 150 cells were counted. **I** Brightfield images of untreated (DMSO) and ceritinib-treated (CERI) cells depict apoptotic cells with active caspase 3/7 (green). Bar graphs represent mean ± SEM of two independent experiments with duplicate measurements. Group comparisons were calculated by student’s t test. All scale bars are adjusted to 100 µm

As the RNA sequencing data suggested nuclear membrane deregulation in tumor samples, we inspected the structural impact of ceritinib treatment on the nuclear membrane by quantifying cells with intact or ruptured nuclear membrane (Fig. 4A). Consistent with gene ontology and pathway enrichment analysis, ceritinib exposure induced nuclear membrane rupture in approximately 80% of cells, whereas control cells largely retained intact nuclei (Fig. 4B). As ceritinib inhibited phosphorylation of MTOR, AKT, FAK, and ERK1/2 (Fig. 2J), we asked whether repression of these kinases could be responsible for the disruption of nuclear membrane architecture. Treatment of tumor cells with the corresponding pharmacological inhibitors led to loss of kinase activity, as evidenced by dephosphorylation of individual kinases (Fig. 4C), but nuclear membranes remained intact (Fig. 4D). These findings suggest that the suppression of kinase activities following ceritinib exposure is not the cause, but rather a consequence of nuclear membrane disruption.

As ceritinib exposure has been reported to induce cellular reactive oxygen species (ROS) levels [28], we next examined ROS generation upon ceritinib treatment. Both fluorescent microscopy and flow cytometry confirmed a substantial increase in ROS levels in ceritinib-treated tumor cells compared to untreated controls (Fig. 4E). Importantly, the ROS pathway was negatively enriched in our patient cohorts, highlighting deregulation of ROS-dependent cell death mechanisms in tumor cells (Fig. 4F). Moreover, immunofluorescence-based evaluation of nuclear morphology revealed that ceritinib exposure significantly decrease nuclear circularity and nuclear area by showing irregular nuclear morphology and nuclear shrinkage of tumor cells, indicating the disruption of nuclear architecture upon ceritinib exposure (Fig. 4G). As the vast majority of cellular ROS is generated from mitochondria [29], we inspected their subcellular localization following ceritinib treatment. Interestingly, we observed drastically elevated perinuclear clustering of mitochondria in cells exposed to ceritinib, in contrast to mitochondria of the control cells displaying a more evenly distributed cytoplasmic distribution (Fig. 4H). Of note, active caspase 3/7 staining of ceritinib-treated tumor cells showed a significant increase of apoptotic cells, suggesting that nuclear membrane disruption and mitochondrial relocalization contributes to programmed cell death (Fig. 4I). Collectively, these findings demonstrate that ceritinib compromises nuclear membrane integrity and causes perinuclear mitochondria accumulation, which consequently promotes apoptosis via cellular ROS generation in pediatric liver tumor cells.

### Ceritinib inhibits tumor cell growth in vitro and in vivo

**Fig. 5:**
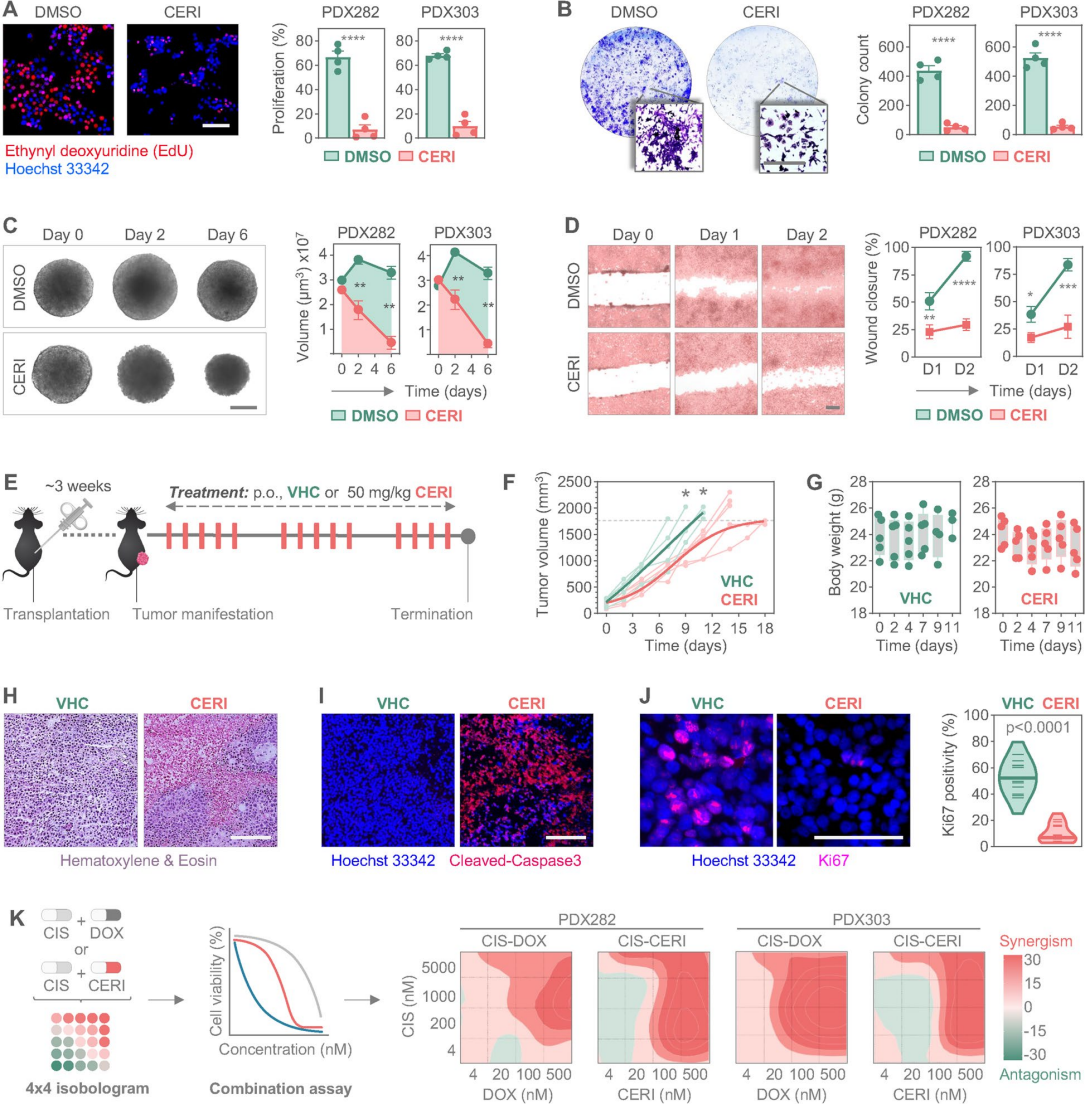
Ceritinib prevents HB tumor growth both in vitro and in vivo*. A* Short-term growth of tumor cells upon control (DMSO) or 250 nM ceritinib (CERI) for 24 h, detected by ethynyldeoxyuridine staining (red) in relation to Hoechst 33342-counterstained nuclei (blue). Representative microscopy images (left) and the portions of proliferating cells (right) are demonstrated. **B** Long-term growth of tumor cells exposed to DMSO or 250 nM CERI for 10 days, determined by colony formation assay. Representative crystal violet-stained wells and magnified views of single colonies are displayed (left). Absolute counts of colonies are given in bar graphs (right). **C** Three-dimensional growth of established tumor spheroids after exposure to DMSO or 250 nM CERI for 6 days, shown as microscopic bright field images (left). Spheroid volumes are given in line graphs for day 0, 2, and 6. **D** Motility of HB tumor cells were monitored by wound healing assay (left), and wound closure rates are given in line graphs (right). **E** Experimental overview of ceritinib treatment in vivo. Immunocompromised mice harboring subcutaneously transplanted PDX282 tumors were treated with 50 mg/kg body weight CERI by oral administration or vehicle (VHC) 5 times per week. Mice were sacrificed when they reached the maximal tolerable tumor size. **F** Tumor growth and (**G**) body weight changes of mice treated with either CERI (*n* = 5) or VHC (*n* = 5). **F** Each light-colored line corresponds to an individual animal and dark-colored lines represent the mean tumor volumes. The dashed line indicates the maximum permissible tumor size. **G** Each dot represents an individual animal. **H** Hematoxylin/eosin staining, **I** immunofluorescent staining of cleaved caspase-3 (red) indicating apoptotic regions, and **J** Ki67-positive nuclei (red) counterstained with Hoechst 33342 (blue) of VHC and CERI-treated tumors. Proliferation rate was calculated by relating Ki67-positive nuclei of three tumors per condition to Hoechst 33342 nuclei and is shown in a violin plot. Short lines represent individual count numbers, long lines indicate the median. **K** Combination assays of cisplatin (CIS), doxorubicin (DOX), and CERI were conducted for 48 h in HB tumor cells and synergies calculated from cell viability data using Synergy Finder +. Two-dimensional contour plots correspond to BLISS synergy scores, with regions of high synergism shown in red. All data shown in graphs are representing the mean ± SEM of two independent experiment with duplicate measurements. Statistics were calculated using a two-tailed unpaired Student’s *t* test, with ns = not significant, **p < 0.01, ***p < 0.001, ****p < 0.0001. All scale bars represent 100 µm

To investigate the effects of ceritinib on the cellular level, we performed immunofluorescence staining, which revealed inhibition of liver tumor cell growth, as evidenced by the significantly lower number of proliferating cells upon ceritinib exposure (Fig. 5A). Moreover, colony formation assays clearly demonstrated the growth inhibitory properties of ceritinib under long-term culturing conditions, with ceritinib-treated models forming fewer colonies than untreated controls (Fig. 5B). To evaluate ceritinib’s efficacy in a more physiologically relevant setting, we tested its effects on established liver tumor spheroids, as three-dimensional models better mimic patient conditions [30]. Ceritinib treatment not only stopped the growth of the tumor spheroids, but also significantly reduced their volume, while control spheres continued to grow (Fig. 5C). To further examine the effect of ceritinib on tumor cell migration, we performed scratch assays. The wound healing capability of tumor cells was notably impaired by ceritinib exposure, with an open scratch gap remaining, while control cells fully closed the wound (Fig. 5D).

To explore the efficacy of ceritinib in a preclinical in vivo setting, we used an established patient-derived xenograft model, in which the highly proliferative and CTNNB1-mutated HB tumor PDX282 was subcutaneous propagated in immunocompromised mice. After an initial latency period of three weeks, mice received oral treatment of either 50 mg/kg body weight of ceritinib or vehicle for 5 days per week over a period of 16 days (Fig. 5E), a regimen that has previously been used safely and efficiently in mice bearing glioblastoma [31]. Ceritinib-treated mice demonstrated a significant reduction in tumor volumes and extended survival in comparison to the vehicle-treated mice (Fig. 5F). Importantly, we did not observe any drastic changes in body weights during the treatment period (Fig. 5G), and no changes in physical appearance and behavior were found. Subsequent histological examination of the treated tumor specimens revealed recurrent areas of eosinophilic remnants and condensed nuclei, which are suggestive of cell death and disruption of tumor tissue integrity (Fig. 5H). Concomitantly, these areas stained positive for cleaved caspase-3, a marker of apoptosis (Fig. 5I). Moreover, immunofluorescent staining of ceritinib-treated tumors displayed a significantly reduced number of Ki67-positive proliferating cells compared to the vehicle-treated tumor tissue (Fig. 5J). Altogether, these results validated the strong growth inhibitory properties of ceritinib in vitro and its safety and efficacy in vivo.

To investigate the potential clinical benefit of combining ceritinib with cisplatin, the current therapeutic backbone for treating HB patients [32], we subjected two HB models to pairwise drug combination experiments (Fig. 5K). A four-point dose–response matrix revealed that the combination of cisplatin and ceritinib achieved already very high synergy scores at nanomolar doses, comparable to those observed with cisplatin and doxorubicin.

## Discussion

Despite significant advancements in the clinical management of hepatoblastoma (HB), there still remains a critical requirement for refined treatment options for patients with high-risk features and refractory disease unresponsive to first-line chemotherapy [32]. Computational tools integrating high-throughput and large-scale sequencing data of patients as well as target interaction and drug inhibition information present promising, safe, and cost-effective approaches for discovering new cancer treatments [33]. However, most drug prediction strategies have been applied to cancers in the adult population. By harnessing digital twins of 13 pediatric liver tumor patients with challenging clinical features and treating them in silico with clinically approved drugs by mechanistic models, we were able to identify and subsequently in vitro and in vivo validate ceritinib as a promising drug for the therapeutic intervention of high-risk pediatric liver tumors.

Mechanistic tumor modeling systems have been designed to personalize precision medicine and accelerate drug development by virtualizing clinical trials. By creating virtual representations of individual tumor cell models, ModCell™ has been designed to predict responses to specific therapies or combinations based on holistic input data [11–13]. We have generated digital twins for 13 pediatric liver tumor patients and trained ModCell™ using transcriptomic profiles, defining Wnt signaling as a key driver of malignancy and MYC as a mechanistic model readout. This approach was then successfully used to mimic treatment strategies for patients by simulating drug responses computationally. ModCell™ predicted several drugs with inhibitory capacities across all samples, but ceritinib emerged as the most selective, demonstrating efficacy in tumor samples while sparing normal liver samples. Subsequent preclinical validation studies further underscored the potential of ModCell™ in identifying promising therapeutic approaches with minimal side effects, suggesting its broader applicability for other pediatric cancers. Moreover, the implementation of ModCell™ for simulating patient response to drug combinations could provide a cost-effective and risk-attenuated approach for determining the optimal combination treatment strategy for pediatric liver tumor patients.

Ceritinib, a second generation ALK inhibitor, was initially described for its potent and selective anti-tumor effects, with nanomolar efficacy in cellular assays [34]. Clinically, it has demonstrated a strong anti-tumor effect as a single drug [35] and superior growth inhibition compared to crizotinib, a first-generation ALK inhibitor [34, 36]. Moreover, ceritinib also displayed activity against lung cancer cell lines that are resistant to alectinib, a third-generation ALK inhibitor [37], suggesting a broader mechanism of action for ceritinib not involving the ALK receptor. Our in vitro testing of different ALK inhibitors confirmed these findings, as liver tumor cell lines demonstrated sensitivity towards ceritinib at very low concentrations. A serendipitous discovery was that ceritinib also displayed anti-proliferative activity in vitro against ALK-negative lung cancer cell lines [23] and demonstrated prominent anti-tumor activity in non-small cell lung cancer patients without ALK rearrangement in clinical trials [38], indicating a broader mechanism beyond ALK inhibition. Consistently, we confirmed growth inhibition of HB cells by ceritinib independent of ALK, as no ALK protein expression was detected, further supporting an ALK-independent mechanism of action for ceritinib. Beyond ALK, ceritinib has been described to inhibit other key regulatory proteins such as AKT, ERK, MTOR and FAK by disrupting phosphorylation [23, 34]. Consequently, our Western blot analysis confirmed that ceritinib deregulates phosphorylation of these kinases, highlighting that ceritinib relies on the simultaneous inhibition of multiple non-canonical targets in pediatric liver cancers.

We further identified the nuclear pore complex (NPC) as another key target of ceritinib exposure in pediatric liver tumor cells, leading to the disruption of nuclear membrane architecture. The nuclear periphery plays crucial roles in maintaining genome stability, as NPCs and nuclear envelope proteins aid transcriptional regulation by interacting with various chromosomal domains [39]. NPCs, composed of around 1,000 protein subunits called nucleoporins (NUPs), serve as the sole gateway between nucleus and cytoplasm and control the nucleocytoplasmic trafficking of macromolecules [40]. Growing evidence suggests that many cancer cells become addicted to nuclear transport, identifying the nuclear transport machinery as both a novel vulnerability of cancer cells and a promising therapeutic target [41]. We identified predominant expression of important NUPs in pediatric liver tumor patients, consistent with the studies indicating that many cancer cells, particularly those resistant to multiple drugs and from aggressive tumors, exhibit increased numbers of NPCs and higher nucleocytoplasmic transport rates, making them reliant on the nuclear transport system [42, 43]. Notably, reducing NPCs in cancer cells has been described to induce death, prevent tumor growth, and result in tumor regression [44]. As none of the pharmacological kinase inhibitors tested in HB cells was able to disrupt nuclear integrity, our data support the conclusion that NUP suppression is a direct effect of ceritinib rather than secondary consequence to kinase inhibition. In line with this, previous studies have reported links between NUPs and kinases, showing that NUP214 and NUP160 modulate ERK and MTOR phosphorylation, respectively [44, 45]. Altogether, we demonstrate that ceritinib prevents formation of NPCs by suppressing the expression of structural NUPs, consequently leading to nuclear membrane rupture in treated tumor cells.

The loss of nuclear membrane integrity has inevitable consequences, as the nucleus in normal conditions is shielded from mitochondrial oxygen-derived free radicals, which could be highly toxic to nuclear DNA [46]. The interplay between the nucleus and mitochondria, via biochemical events including reactive oxygen species (ROS) and Ca^2+^ transport mechanisms, is very important for the nuclear stabilization of pro-survival transcriptional factors, which are highly active in malignancies [47]. Moreover, mitochondria can establish novel contact sites with mitochondria under stress conditions, leading to perinuclear accumulation of mitochondria and subsequent nuclear exposure to oxygen radicals [46]. Consistent with previous findings on high-grade ovarian carcinoma [28], we observed elevated cellular ROS levels upon ceritinib exposure. Furthermore, we identified that ceritinib promotes perinuclear clustering of mitochondria and triggers apoptosis. Considering that the physical separation between the nucleus and the cytoplasm is corrupted by ceritinib due to nuclear membrane disruption, nuclei of cancer cells are highly vulnerable to perinuclear localization of mitochondria that expose toxic radicals to genomic material.

While our findings provide strong preclinical evidence supporting ceritinib as a potential therapy, additional studies are necessary to fully elucidate its mechanisms of action, particularly its effects on non-canonical kinases and nuclear integrity. Further validation in larger patient cohorts and clinical trials will be required to determine its clinical efficacy, optimal dosing strategies, and potential combination therapies. Based on our data it is tempting to speculate that ceritinib could potentially be used to partially substitute for or even replace doxorubicin in the clinical setting. Additionally, the applicability of mechanistic modeling to other pediatric cancers should be explored to expand the scope of digital twin-based drug discovery. Selecting appropriate model conditions appears critical, as our pilot simulations incorporating CTNNB1 alongside MYC:MAX and Caspase-3 or GLI1 as a readout of Hedgehog activation in ModCell™ did not yield effective or only subset-specific drug candidates.

Collectively, by adopting ModCell™ to the use of virtual pediatric liver tumor patient cell models with high-risk features we were able to define ceritinib as the most promising treatment option. Computational predictions were further validated through multilayered validation in vitro and in vivo studies. Our study established ceritinib as a multifaceted compound that simultaneously interferes with various signaling pathways activated in pediatric liver cancers, independent of ALK expression. This work strengthens the potential of machine-learning based technologies for precise, safe, and effective drug prediction and repositioning in the pursuit of cancer treatment.

## Conclusions

In conclusion, this study demonstrates the potential of mechanistic models and virtual patient simulations in identifying effective therapies for high-risk pediatric liver tumors. By integrating multi-dimensional clinical and molecular data to generate digital twins, we identified ceritinib as a promising treatment candidate. Its efficacy was validated in vitro and in vivo, revealing potent anti-tumor activity through novel mechanisms involving disruption of nuclear integrity. These findings support ceritinib as a targeted therapeutic option and underscore the value of computational modeling in guiding precision oncology.

## Supplementary Information

Supplementary Material 1.

## Data Availability

Data are available at Gene Expression Omnibus (accession number GSE306477).
